# Mechanism of salidroside against coronary artery disease by network pharmacology analysis

**DOI:** 10.1186/s12906-023-04027-3

**Published:** 2023-06-12

**Authors:** Lin Tao, Zhi-Fang Liang, Liu Miao, Yu-Jie Guo, Ye Li, Yan-Li Liu, Dong-Ming Fang, Zhi-Jie Yang

**Affiliations:** Departments of Cardiology, Liuzhou People’s Hospital, 8 Wenchang Road, Liuzhou, 545006 Guangxi People’s Republic of China

**Keywords:** Gene expression omnibus, Salidroside, Coronary artery disease, Network pharmacological

## Abstract

**Background:**

Rosenroot (Rhodiola rosea) is a traditional Chinese herbal medicine. It has been used to treat patients with coronary artery disease (CAD). Salidroside is the main active constituent of rosenroot. This study was designed to explore the mechanism of salidroside in treating CAD and its role in angiogenesis in CAD systematically.

**Methods:**

In this study, potential targets related to salidroside and CAD were obtained from public databases. Gene Ontology (GO), Kyoto Encyclopedia of Genes and Genomes (KEGG), Disease Ontology (DO) and CellMarker enrichment analyses were performed. The binding of salidroside to angiogenesis-related targets was assessed by PyMOL and Ligplot. Furthermore, the effects of salidroside on collateral circulation were evaluated by correlation analysis of these angiogenesis-related targets with the coronary flow index (CFI), and the influence of salidroside on human umbilical vein endothelial cell (HUVEC) proliferation and migration was assessed.

**Results:**

Eighty-three targets intersected between targets of salidroside and CAD. GO and KEGG analyses indicated that salidroside mainly treated CAD through angiogenesis and anti-inflammatory action. There were 12 angiogenesis-related targets of salidroside in coronary heart disease, among which *FGF1* (r = 0.237, *P* = 2.597E-3), *KDR* (r = 0.172, *P* = 3.007E-2) and *HIF1A* (r = -0.211, *P* = 7.437E-3) were correlated with the coronary flow index (CFI), and salidroside docked well with them. Finally, cell experiments confirmed that salidroside promoted the proliferation and migration of HUVECs.

**Conclusions:**

This study revealed the potential molecular mechanism of salidroside on angiogenesis in CAD and provided new ideas for the clinical application of salidroside in the treatment of CAD.

**Supplementary Information:**

The online version contains supplementary material available at 10.1186/s12906-023-04027-3.

## Background

Coronary artery disease (CAD), also known as coronary atherosclerotic heart disease, is a principal cause of cardiovascular death globally, and the mortality rates of CAD are increasing annually [1]. Percutaneous coronary intervention (PCI) and coronary artery bypass grafting (CABG) have greatly improved the symptoms and prognosis of CAD. However, certain patients cannot be treated surgically because of diffuse coronary artery disease [2]. Currently, effective angiogenesis and coronary collateral circulation are critical as they curtail cardiovascular events and ameliorate prognosis [3, 4].

Rosenroot (*Rhodiola rosea*) is a traditional Chinese herbal medicine and has significant medicinal and health benefits [5]. It has been extensively employed for numerous diseases, such as coronary artery diseases, hypertension, heart failure, heart arrhythmia and other cardiovascular diseases [6, 7]. Salidroside, one of the key active constituents of rosenroot, has been synthesized for clinical treatment and basic research. It plays an important role in the cardiovascular system due to its anti-hypoxia and antioxidant activities, antiaging activities, anti-inflammatory and antidiabetic activities [8]. It has been reported that salidroside inhibits ox-LDL-induced human coronary artery endothelial cell apoptosis by regulating miR-133a/Bcl-XL signaling, thereby preventing atherosclerosis progression [9]. Moreover, salidroside can suppress autophagy by upregulating circ-0000064, which enhances cardiac function and reduces the area of myocardial infarction [10].

**Fig. 1:**
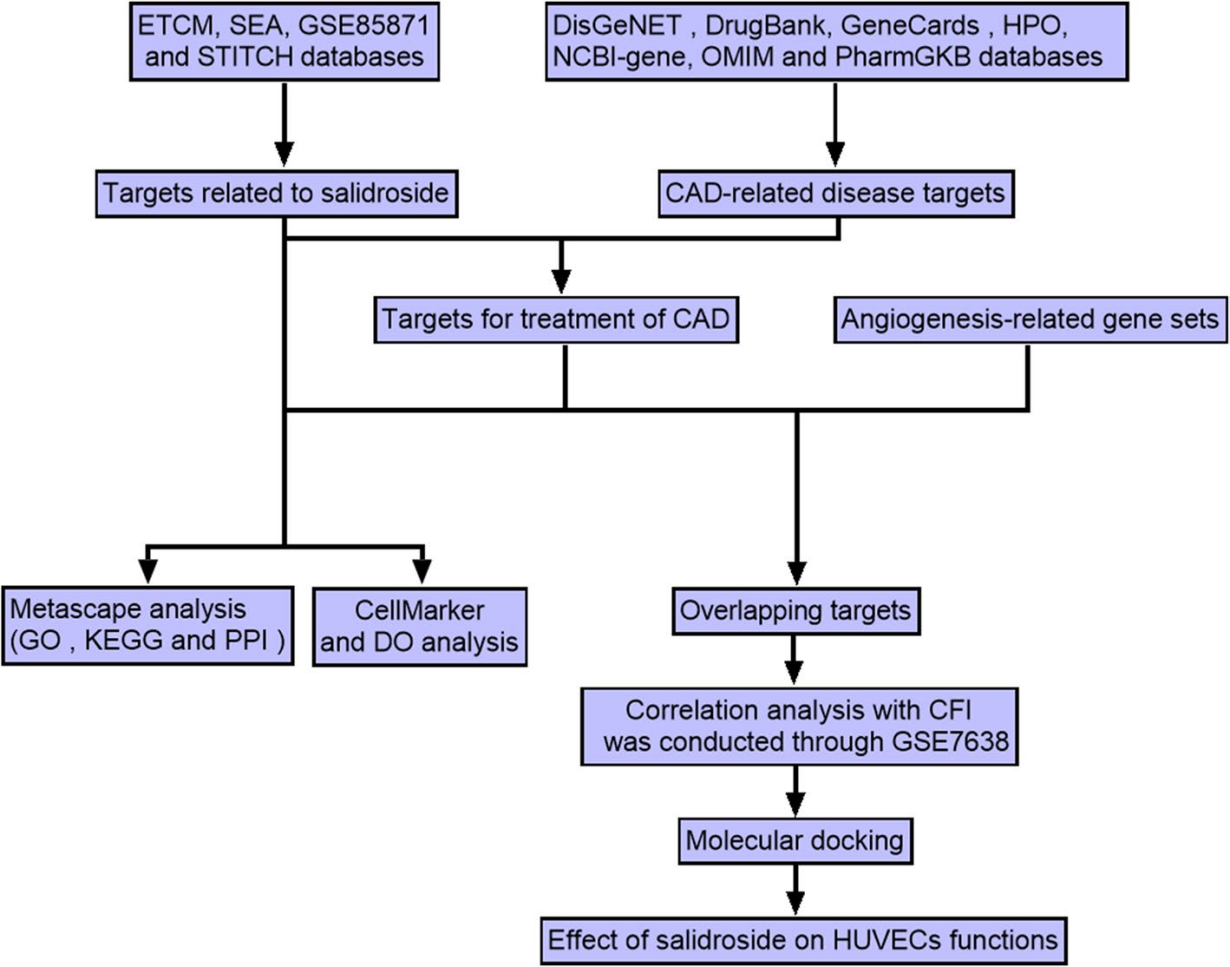
Flowchart of the design

Salidroside is an effective drug for the treatment of CAD, but its mechanism of action on angiogenesis and collateral circulation is still unclear. Network pharmacology is a field of drug interaction modeling using computer technology and bioinformatics, which can accelerate the screening and analysis of active substances, targets, and molecular mechanisms of traditional medicine [11, 12]. The aim of this study was to investigate the mechanism of salidroside in the treatment of CAD and its effect on angiogenesis and collateral circulation by network pharmacology and experimental methods. The analysis process is shown in Fig. 1.

## Methods

### Structure and pharmacokinetic properties of salidroside

Using “salidroside” as the search term, the PubChem database (https://pubchem.ncbi.nlm.nih.gov/) was used to find the chemical structure of salidroside. The TCMSP database (https://tcmsp-e.com/) was used to evaluate the pharmacokinetic properties of salidroside, such as oral bioavailability (OB), Caco-2 permeability (Caco-2), and blood–brain barrier (BBB).

### Obtaining potential targets related to salidroside

To identify the potential targets for salidroside, the Encyclopedia of Traditional Chinese Medicine (ETCM, http://www.tcmip.cn/ETCM/), similarity ensemble approach (SEA, https://sea.bkslab.org/) and STITCH (http://stitch.embl.de/) databases were used. Concurrently, dataset GSE85871 was obtained from the Gene Expression Omnibus (GEO, https://www.ncbi.nlm.nih.gov/geo/) database. This dataset contains RNA sequencing (RNA-seq) data from MCF-7 cells treated with 102 different herbs, including salidroside, to help investigate the potential pharmacological mechanisms and molecular targets of the various herbs [13]. After normalization between arrays, differentially expressed genes (DEGs) were analyzed through the limma package of R. DEGs were defined as genes with |log2-fold change |> 1 and a P value < 0.05. Then, the results of DEGs, ETCM, SEA and STITCH databases were merged to obtain salidroside related targets.

### Obtaining potential targets related to CAD

To obtain potential targets associated with CAD, we searched for DisGeNET (https://www.disgenet.org/) with a score > 0.2, DrugBank (https://www.drugbank.com/), GeneCards (https://www.genecards.org/) with score > 20, The Human Phenotype Ontology (HPO, https://hpo.jax.org/app/), NCBI-gene (https://www.ncbi.nlm.nih.gov/gene/), Online Mendelian Inheritance in Man (OMIM, https://omim.org/) and PharmGKB (https://www.pharmgkb.org/) databases using “coronary artery disease”,” coronary heart disease”,” Ischemic heart disease”,” coronary disease” and “coronary occlusion” as search terms. All database results were combined to obtain the CAD-related disease targets.

### Metascape analysis

Metascape (https://metascape.org) is a powerful online bioinformatics analysis tool that integrates Gene Ontology (GO), Kyoto Encyclopedia of Genes and Genomes (KEGG) [14–16], The Universal Protein Resource (UniProt) and many other authoritative data resources [17]. The intersection of CAD-related targets and salidroside-related targets was used to obtain salidroside-related targets for the treatment of CAD. Then, salidroside-related targets and targets for the treatment of CAD were submitted to Metascape, with the species limited to “*Homo sapiens*”. GO, KEGG and protein–protein interaction (PPI) network analyses were performed. Meanwhile, key modules were identified by the default algorithm of Metascape. Terms with adjusted P values < 0.05 were collected. Finally, the results were visualized using the ggplot2 package in R software and Cytoscape.

### CellMarker and DO analysis

Salidroside-related targets and targets for the treatment of CAD were further used for CellMarker and DO Analysis. The CellMarker database (http://xteam.xbio.top/CellMarker/) contains 13,605 cell markers of 467 cell types in 158 human tissues/subtissues and 9148 cell markers of 389 cell types in 81 mouse tissues/subtissues. Marker genes expressed in tissues and cells can be found through the CellMarker database [18]. First, target names were converted into Entrez IDs, then “Human cell markers.txt” was downloaded from the CellMarker database, and the “clusterProfiler” package was used for cell and tissue enrichment analysis. Finally, DO analysis was performed by the “DOSE” package.

### Acquisition of angiogenesis-related gene sets

Gene sets associated with angiogenesis were detected using the Molecular Signatures Database (MSigDB) [19, 20] with "angiogenesis" as the search term and excluded all species other than humans.

### Identifying genes related to angiogenesis in salidroside treatment of CAD

To identify the angiogenesis-related genes in salidroside treatment of CAD by crossing the angiogenesis-related genes with the targets of salidroside treatment of CAD. Further, Pearson correlation analysis was performed using the RMA normalized matrix of GSE7638 to evaluate the association between these genes and coronary flow index (CFI) [21].

### Molecular docking

The protein structures of genes related to angiogenesis in salidroside treatment of CAD were downloaded from the Protein Data Bank (http://www.rcsb.org/pdb/) [22]. The water molecules were deleted from the protein structure, hydrogen atoms were added, and Gasteiger charges were calculated. Additionally, the ligand structures must conform to the low-energy conformation. Moreover, the box size and coordinates in molecular docking were finally determined based on ligand position. Then, AutoDock Vina software was used to perform blind docking between salidroside and intersection targets with the exhaustiveness parameter was set at 8 and the energy range parameter was set at 5. Thereafter, the ligand was integrated into the target proteins in a semi-flexible manner, resulting in a total of 20 conformations. The most affinal conformation was chosen to be the eventual docking conformation [23]. Pymol2.4 and Ligplot2.2 were used to further analyze their binding patterns, binding affinity and critical interactions.

#### Impact of salidroside on the proliferation of HUVECs

Human umbilical vascular endothelial cells (HUVECs; #GDC166, CCTCC) were seeded in 96-well plates (Costar, Cambridge, MA, USA) at 3000 per well. On Day 2, the medium was exchanged for serum-free medium for 10 h. Then, the control group was cultured with complete medium, and the salidroside group was cultured with complete medium containing 30 μM salidroside. After 24 h, cell proliferation rates were subsequently assessed using the cell counting kit-8 (CCK-8, UElandy, China) according to the manufacturer’s instructions. The optical density (OD) values at 450 nm and 570 nm were measured. The OD value at 450 nm was subtracted from the OD value at 600 nm to obtain the corrected OD value of each well.

### Impact of salidroside on the migration of HUVECs

A total of 300000 cells/well were plated into a 6-well plate (Costar, Cambridge, MA, USA) and incubated to reach confluence. The monolayer was scratched using a 20 μl tip and washed with phosphate buffered saline to remove detached cells. The cells were cultured in serum-free medium supplemented with or without salidroside (30 μM). An inverted microscope (magnification, × 40; IX71; Olympus Corporation) was used to observe the healing of the scratches 24 h later, and the results were treated with ImageJ.

### Statistical Analysis

All experiments were independently repeated 3 times. Statistical analysis was carried out using the GraphPad Prism 7.0 statistical software package, and *P* < 0.05 was considered statistically significant.

## Results

### The structure and pharmacokinetics properties of salidroside

**Fig. 2:**
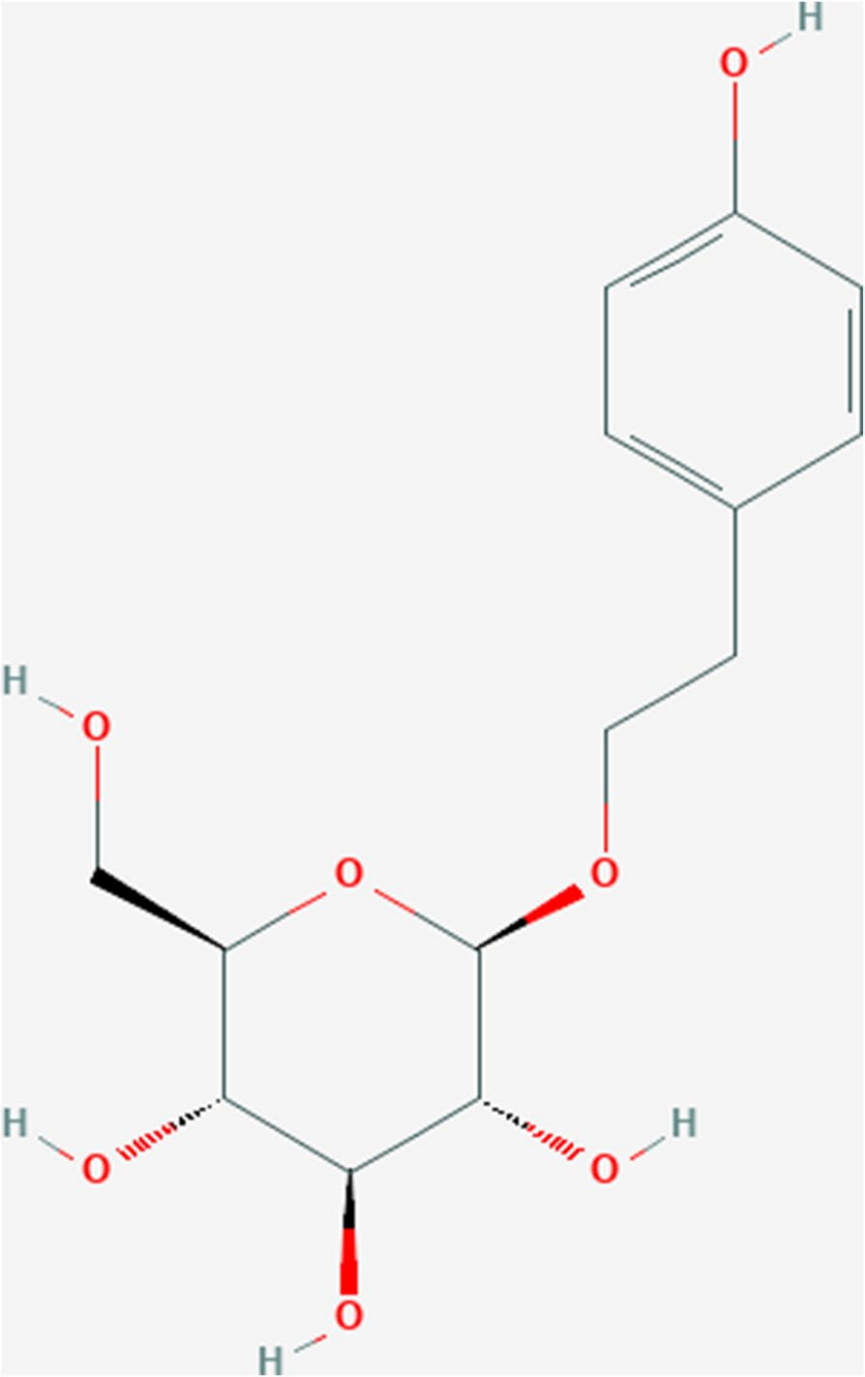
Chemical structure depiction of salidroside (CID:159278)

The 2D structure of salidroside was downloaded from PubChem (Fig. 2), and the PubChem CID of salidroside was 159278. Further TCMSP analysis of salidroside revealed that the OB of salidroside was 7.01%, that of Caco-2 was -0.82, and that of BBB was -1.41. The detailed pharmacokinetic properties of salidroside are shown in Table 1.

**Table 1:** Pharmacological and molecular properties of salidroside

Name	MW	AlogP	Hdon	Hacc	OB (%)	Caco-2	BBB	DL	FASA	TPSA	RBN
Salidroside	300.34	-0.47	5	7	7.01	-0.82	-1.41	0.2	119.61	0.3	5

*Abbreviations*: *Caco-2* Caco-2 permeability, *OB* oral bioavailability, *DL*, drug likeness, *BBB* blood–brain barrier

### Potential targets of salidroside and CAD

**Fig. 3:**
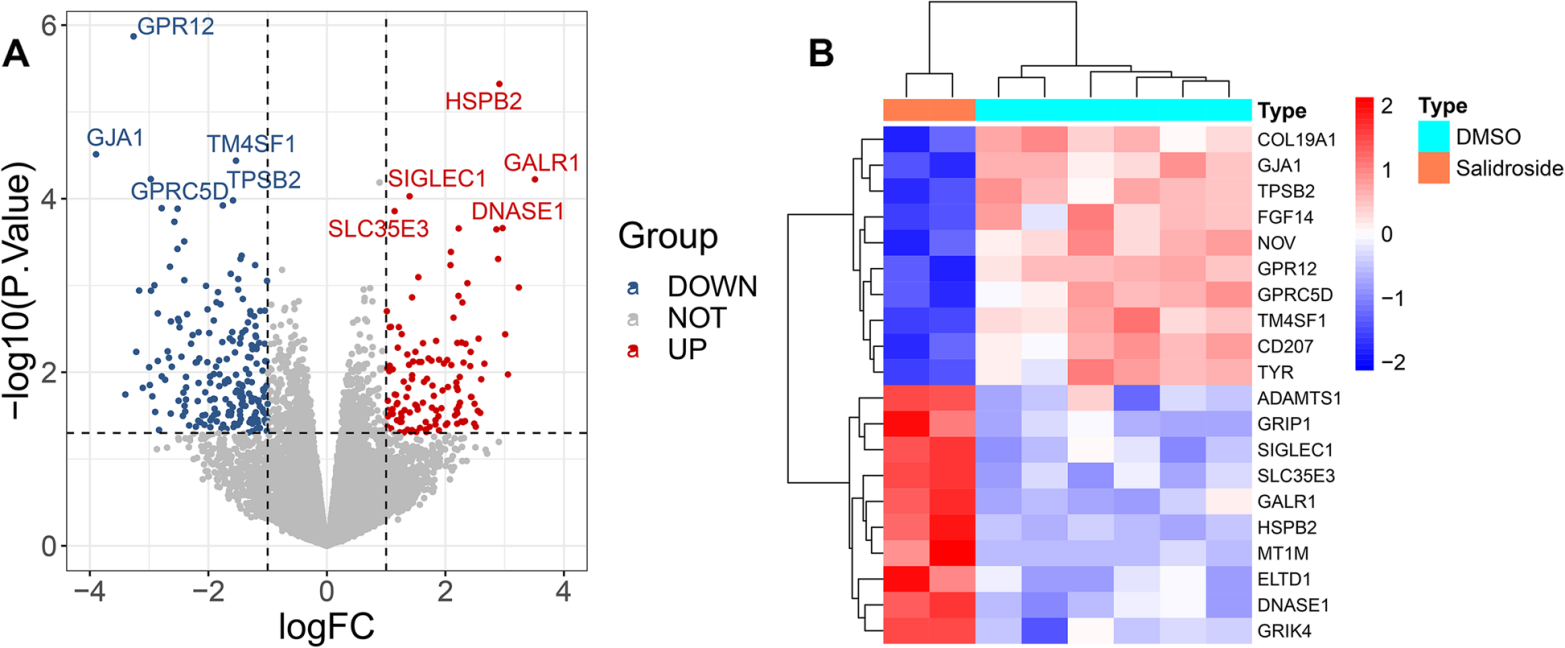
Volcano plot and heatmap displaying the DEGs between the DMSO and salidroside groups. **A** Volcano plot of the DEGs. **B** Heatmap of the top 20 genes with the most significant upregulation and downregulation

**Fig. 4:**
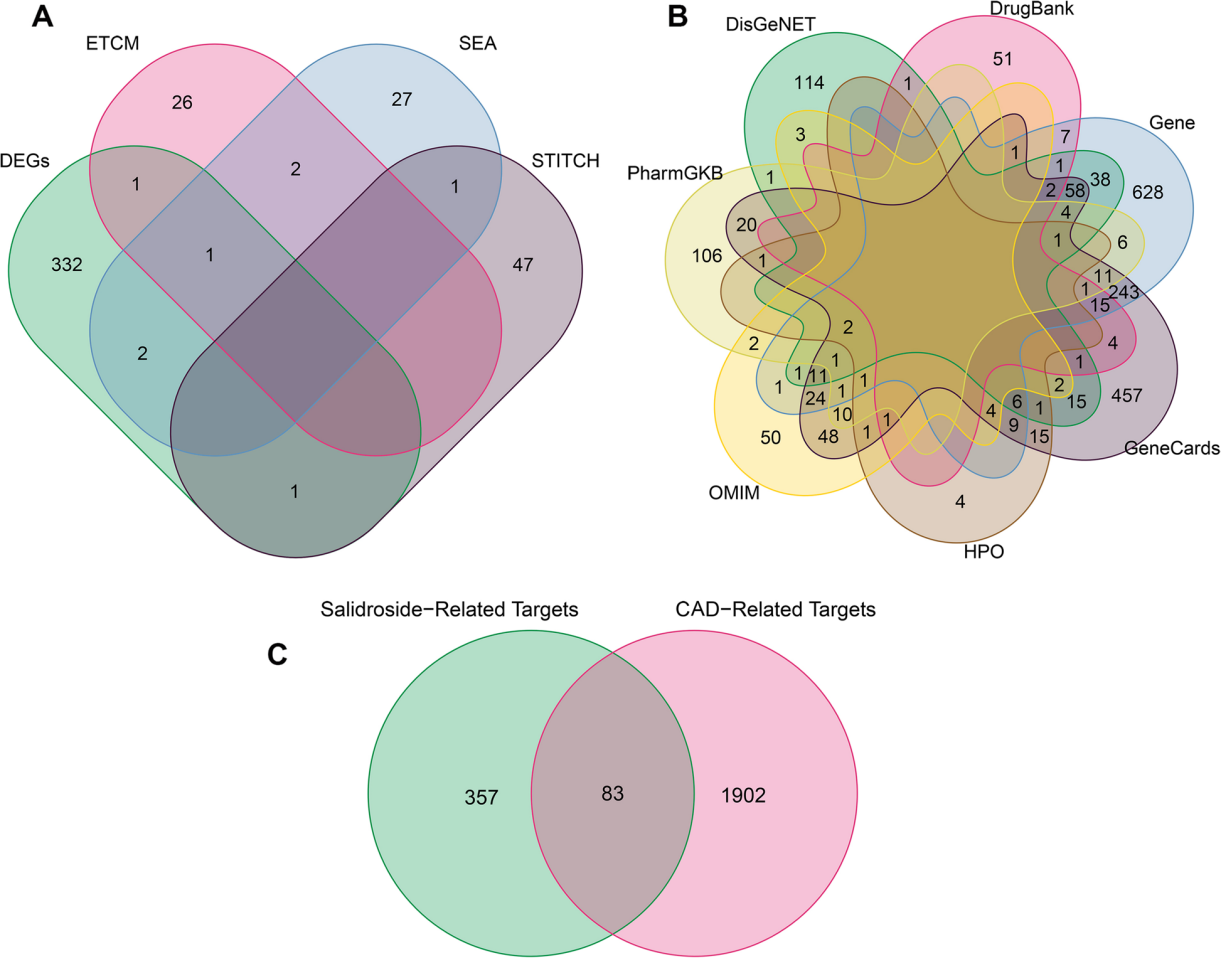
Venn analysis. **A** Union of salidroside-related targets. **B** Union of CAD-related targets. **C** Eighty-three overlapping targets between salidroside-related targets and CAD-related targets

A differential gene analysis was performed on the control and salidroside groups, and the results showed that there were 337 DEGs, of which 130 were upregulated and 207 were downregulated in the salidroside groups. The DEGs are presented in volcano plots and heatmaps (Fig. 3). By screening the ETCM, SEA and STITCH databases, 30, 33 and 49 targets related to salidroside were obtained, respectively. After all data deduplication, 440 potential targets were retained (Fig. 4). To obtain the CAD-related disease targets, the retrieved results of multiple disease databases were integrated. After merging the results of 7 databases and removing duplicates, a total of 1985 potential targets were finally obtained (Supplementary Table 1).

### Enrichment analysis of salidroside-related targets and targets for treatment of CAD

**Fig. 5:**
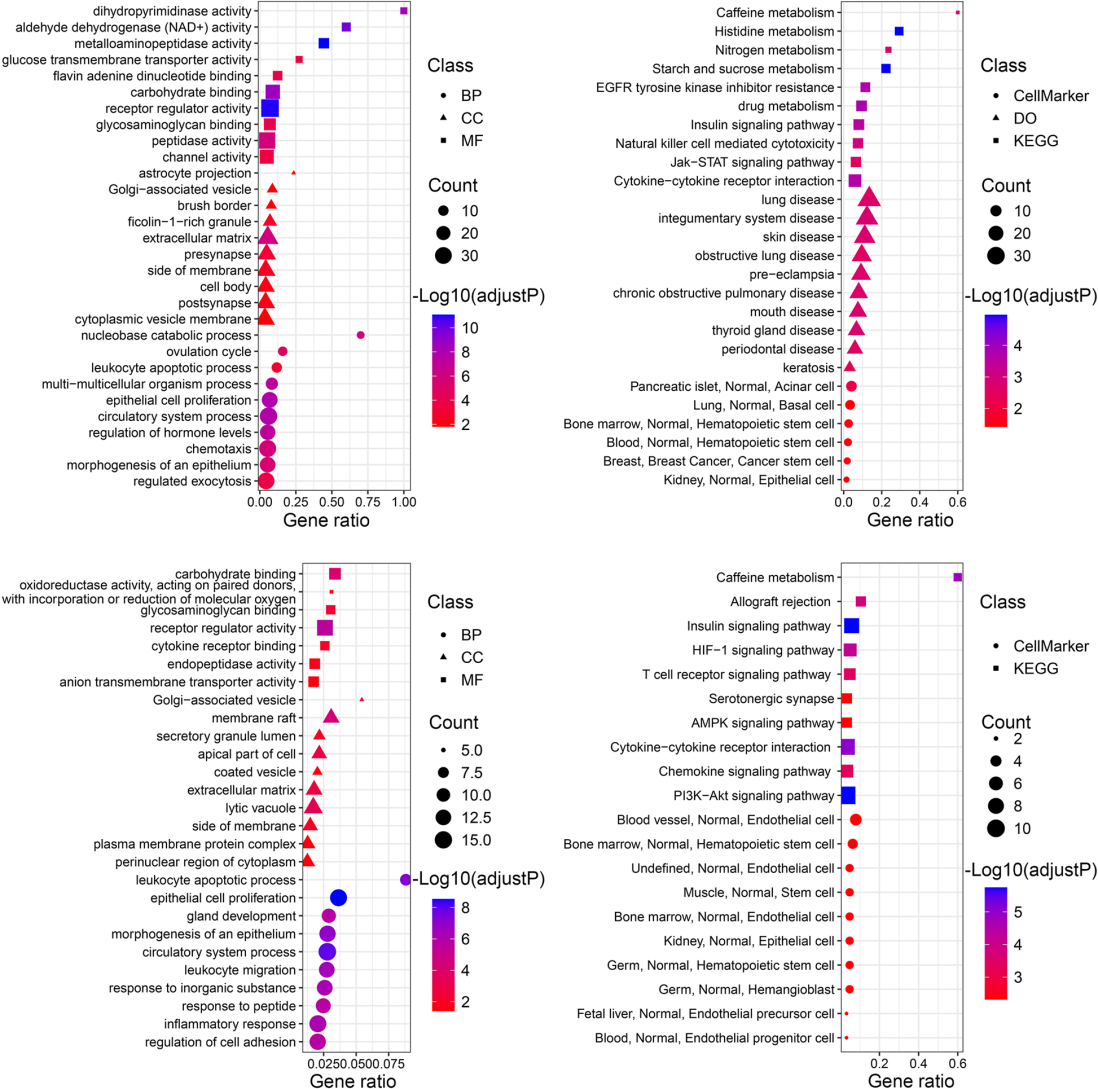
GO, CellMaker, DO and KEGG pathway analyses. **A**, **B** Targets of salidroside. **C**, **D** Targets of salidroside in the treatment of CAD

**Fig. 6:**
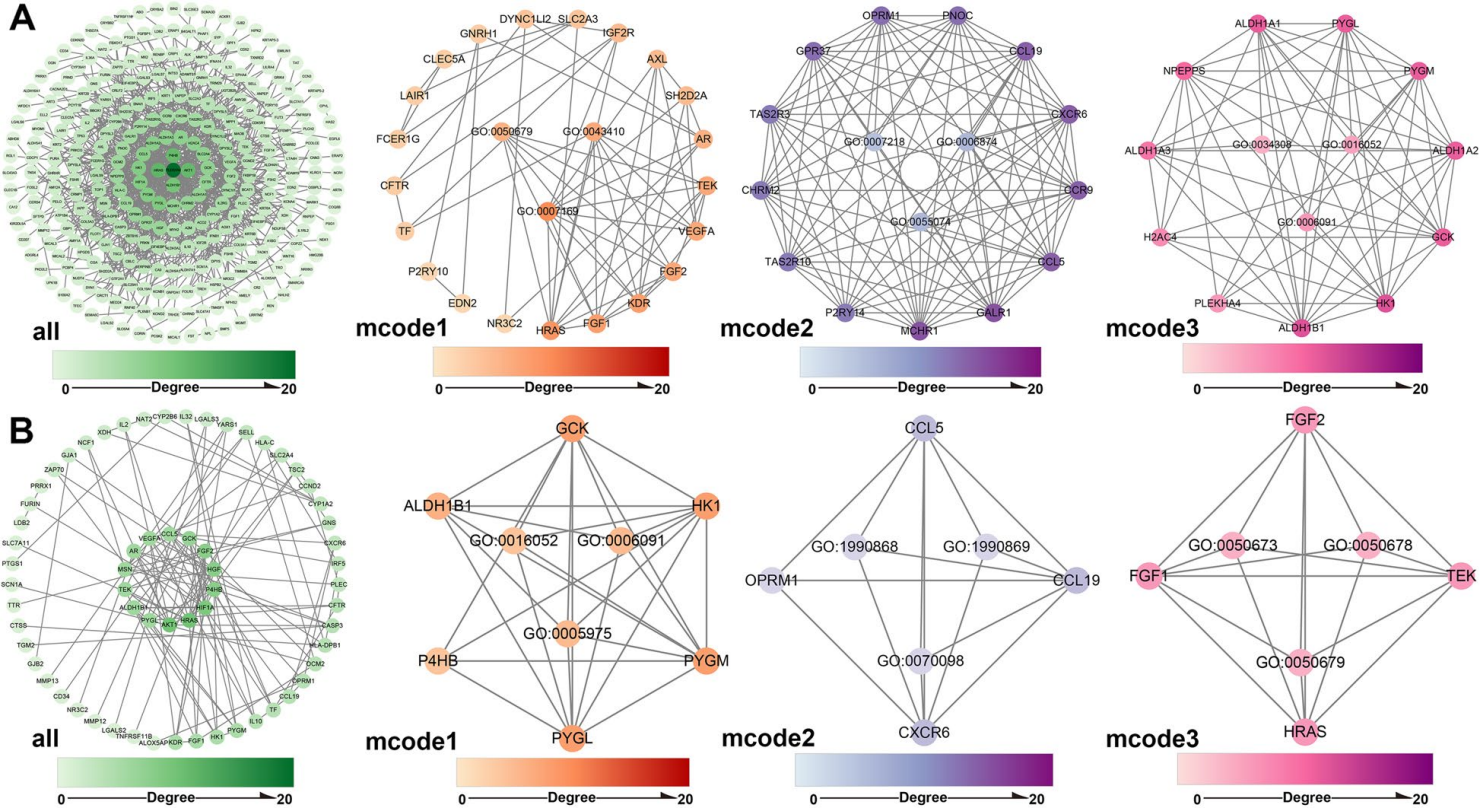
Protein–protein interaction analysis. **A** Targets of salidroside and the key modules. **B** Targets of salidroside in the treatment of CAD and the key modules. The depth of the color represents the degree of correlation, and the deeper the color is, the higher the correlation

Through the Metascape online analysis tool, many GO terms and KEGG terms related to salidroside-related targets and targets for the treatment of CAD were enriched (Fig. 5), and the PPI network and key modules between the targets were obtained (Fig. 6). Interestingly, both salidroside-related targets and targets for the treatment of CAD were enriched in the biological processes of epithelial cell proliferation and morphogenesis of the epithelium. In addition to key module analysis results, there was still a high correlation with epithelial cell proliferation (Table 2). Further CellMarker enrichment and disease ontology analyses were performed by R (Fig. 5). The detailed results can be found in Supplementary Table 2. Notably, CellMarker enrichment analysis of the targets for the treatment of CAD revealed that these targets were enriched in blood vessels, bone marrow, muscle and other normal tissues. It was also enriched in endothelial cells and endothelial progenitor cells.

**Table 2:** Top 3 biological processes of Top 3 modules between targets of salidroside and targets of salidroside in the treatment of CAD

	Module	Term	Description	-Log10(adjustP)
Targets of salidroside	MCODE_1	GO:0007169	transmembrane receptor protein tyrosine kinase signaling pathway	7.4
	MCODE_1	GO:0050679	positive regulation of epithelial cell proliferation	7.2
	MCODE_1	GO:0043410	positive regulation of MAPK cascade	6.2
	MCODE_2	GO:0007218	neuropeptide signaling pathway	6.5
	MCODE_2	GO:0006874	cellular calcium ion homeostasis	5.5
	MCODE_2	GO:0055074	calcium ion homeostasis	5.4
	MCODE_3	GO:0016052	carbohydrate catabolic process	5.8
	MCODE_3	GO:0006091	generation of precursor metabolites and energy	5.6
	MCODE_3	GO:0034308	primary alcohol metabolic process	5.3
Targets of salidroside in the treatment of CAD	MCODE_1	GO:0006091	generation of precursor metabolites and energy	5.4
	MCODE_1	GO:0005975	carbohydrate metabolic process	5.1
	MCODE_1	GO:0016052	carbohydrate catabolic process	5.1
	MCODE_2	GO:0070098	chemokine-mediated signaling pathway	4.7
	MCODE_2	GO:1,990,869	cellular response to chemokine	4.6
	MCODE_2	GO:1,990,868	response to chemokine	4.6
	MCODE_3	GO:0050679	positive regulation of epithelial cell proliferation	5.9
	MCODE_3	GO:0050678	regulation of epithelial cell proliferation	5.1
	MCODE_3	GO:0050673	epithelial cell proliferation	4.9

### Salidroside and angiogenesis

**Fig. 7:**
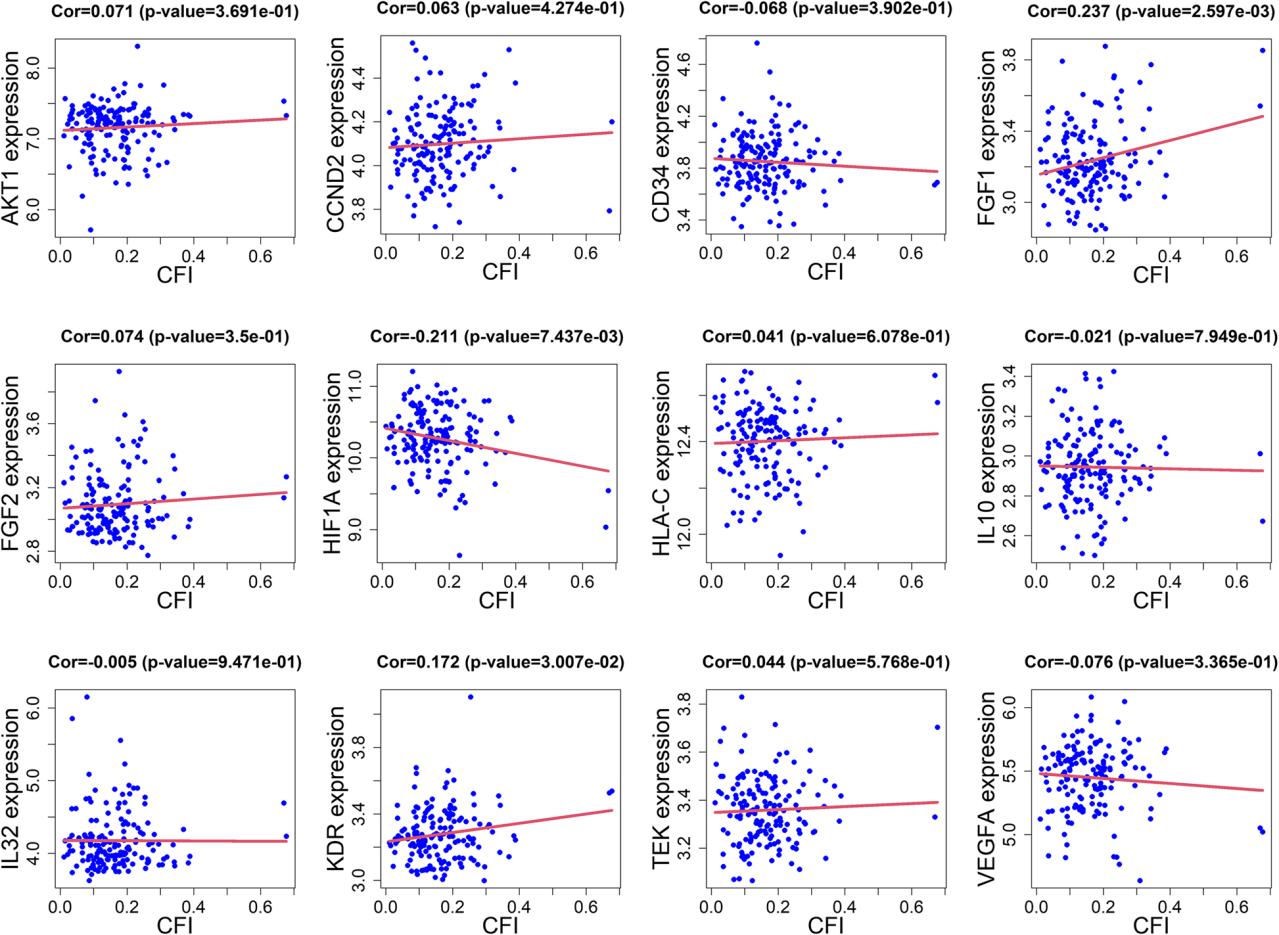
Correlation between 12 angiogenesis-related genes and coronary flow index (CFI) in GSE7638

**Fig. 8:**
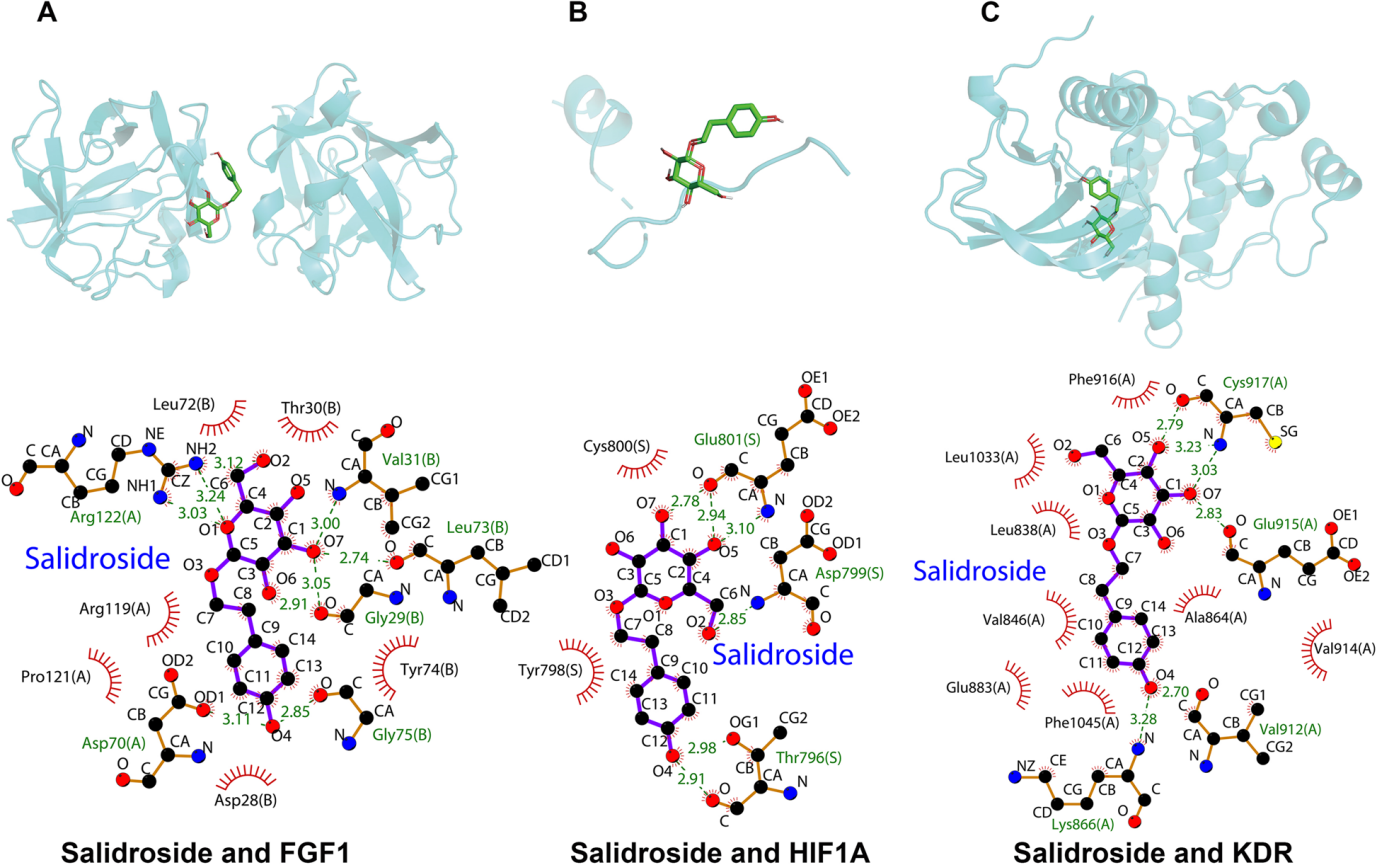
Molecular models of salidroside binding to angiogenesis-related proteins. **A** Docking mode and interactions between salidroside and FGF1, (**B**) salidroside and HIF1A, (**C**) salidroside and KDR. Salidroside is shown in green, red spoke arcs represent hydrophobic contacts, and the green dashed line represents hydrogen bonds

Twenty-five gene sets related to angiogenesis were found in misgdb, containing a total of 463 genes (Supplementary Table 3). After intersecting the angiogenesis-related genes and the target of salidroside in the treatment of CAD, 12 genes remained, including *VEGFA, KDR, CD34, FGF2, AKT1, FGF1, IL10, TEK, CCND2, IL32, HLA-C, and HIF1A*. To further evaluate the binding affinity of these targets and salidroside, molecular docking analysis was conducted. However, the protein structures of *CD34* and *IL32* were not found in the Protein Data Bank, so they were excluded. The binding energies of salidroside and target proteins are shown in Table 3. The results showed that the docking energy of salidroside with the protein receptor ranged from -3.8 kcal/mol to -8.4 kcal/mol. These results suggested that salidroside docked well with these angiogenesis-related proteins under natural conditions. In particular, salidroside presented the lowest docking energy with AKT1 (docking energy: -8.4 kcal/mol). Furthermore, the correlation analysis showed that CFI was positively correlated with *FGF1* (r = 0.237, *P* = 2.597E-3) and *KDR* (r = 0.172, *P* = 3.007E-2) but negatively correlated with *HIF1A* (r = -0.211, *P* = 7.437E-3). The detailed results are shown in Fig. 7. Finally, molecular docking results of salidroside with FGF1, KDR and HIF1A were visualized using pyMOL and Ligplot (Fig. 8). Salidroside mainly binds to the targets by forming multiple hydrogen bonds and hydrophobic interactions with the amino acid residues.

**Table 3:** The docking scores of salidroside with angiogenesis-related proteins

Target	PDB ID	Binding energy (kcal/mol)
AKT1	6S9W	-8.4
KDR	1Y6B	-7.2
TEK	2OO8	-7.2
VEGFA	2VPF	-7.1
HLA-C	1IM9	-7.0
FGF1	1JY0	-6.6
FGF2	1BAS	-5.8
IL10	1ILK	-5.8
HIF1A	1H2L	-4.5
CCND2	6EI2	-3.8

### Salidroside promotes the proliferation and migration of HUVECs

**Fig. 9:**
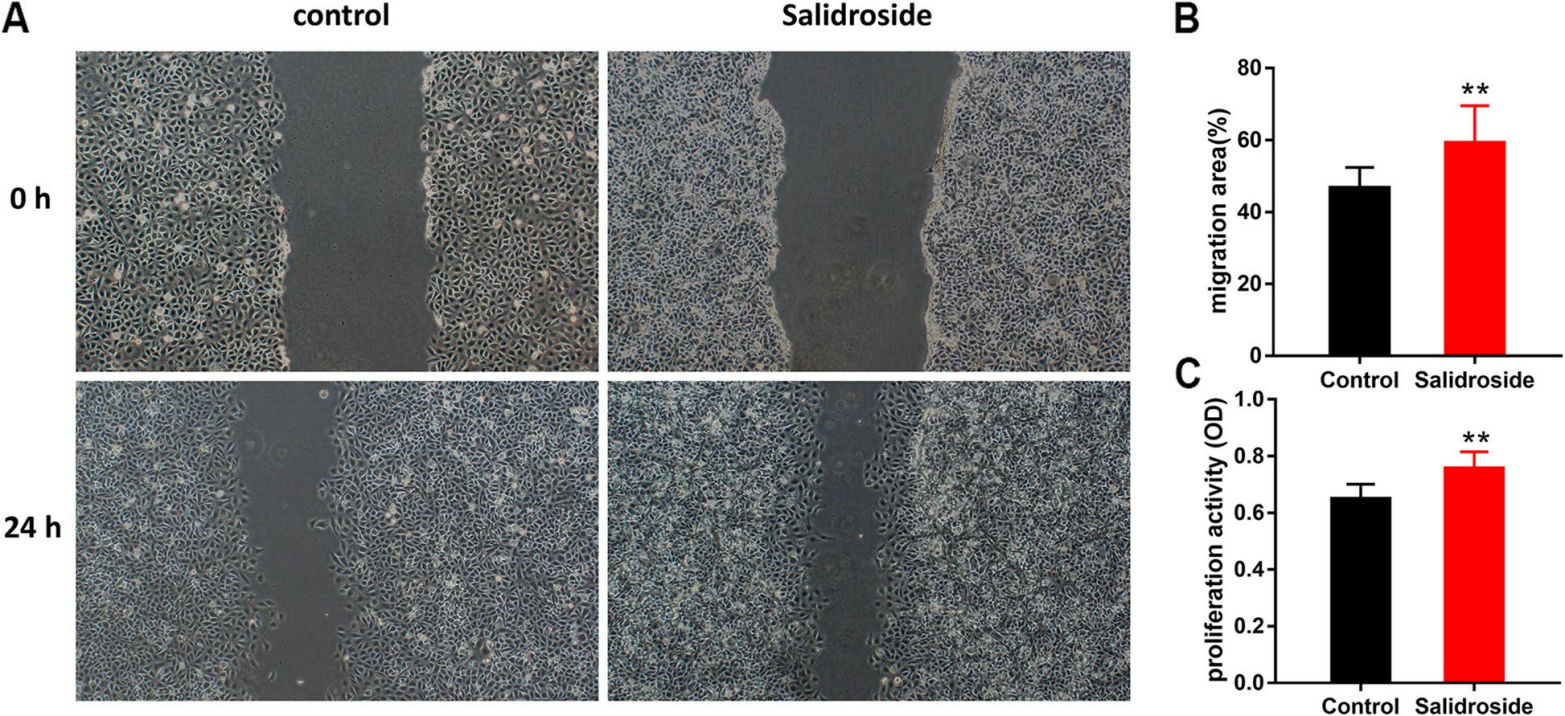
Salidroside promotes the proliferation and migration of HUVECs. **A**, **B** Wound healing assay was performed in HUVECs between different groups. **C** Proliferation activity of HUVECs was detected by cell counting Kit-8 (CCK8) assay. Error bars represented as the mean ± SD. ** indicated *P* < 0.01 versus control

To verify the effect of salidroside on HUVECs, CCK-8 and wound healing assays were performed. Our results indicated that 30 μM salidroside significantly increased the capacity to proliferate and migrate HUVECs compared with the control group (*P* < 0.01, Fig. 9). These results suggested that salidroside enhanced endothelial cell proliferation and migration and angiogenesis.

## Discussion

The pathophysiological mechanisms of CAD are complex. It is well known that CAD is a chronic inflammatory disease. Several proinflammatory cytokines, such as IL-6, IL-18, TNF-α and C-reactive protein, have been reported to be independently associated with the risk of coronary heart disease [24, 25]. In atherosclerotic lesions, inflammatory cells such as T cells, macrophages and mast cells release various inflammatory cytokines and proteolytic enzymes. Phenotypic transformation of vascular smooth muscle cells (VSMCs) is then induced, leading to the proliferation and migration of VSMCs and ultimately promoting the progression of atherosclerotic lesions [26]. Thus, some clinical trials have also demonstrated that anti-inflammatory agents such as colchicine and interleukin-1β antagonists can reduce cardiovascular events and decrease mortality in patients with CAD [27, 28]. In addition to the inflammatory response, oxidative stress, hypoxia and other mechanisms promote the occurrence and development of CAD synergistically.

The enrichment analysis of salidroside in the treatment of CAD suggests that salidroside can regulate inflammatory biological processes, such as leukocyte apoptotic process, leukocyte migration, and inflammatory response in the treatment of CAD, which is consistent with the pharmacological mechanism of salidroside [8]. Salidroside may inhibit the formation of ox-LDL-induced THP1-derived foam cells by inhibiting oxidative stress and the inflammatory response, thus playing an antiatherosclerotic role [29]. In addition, salidroside has a protective effect on myocardial injury in coronary artery occlusion-induced rats by regulating AMPK-related signaling cascades and inhibiting excessive release of myocardial enzymes and proinflammatory cytokines [30].

More intriguingly, many biological processes and signaling pathways related to angiogenesis were enriched in the enrichment analysis. Angiogenesis is the formation of new blood vessels. In the process of angiogenesis, endothelial cells proliferate, migrate and assemble to form tubular structures and then recruit smooth muscle cells and parietal cells to form mature blood vessels [31, 32]. High angiogenic capacity can promote the formation of coronary artery collateral branches, which improves cardiac circulation and myocardial contractility and reduces the incidence of adverse vascular events and mortality. Heeschen et al. reported that angiogenesis-related factors vascular endothelial growth factor (VEGF) and hepatocyte growth factor (HGF) are independent prognostic factors in patients with acute coronary syndrome, and HGF elevation is associated with improved collateralization [33]. Secretoneurin has been reported to increase VEGF-induced VEGFR2 activation and VEGF binding to human coronary artery endothelial cells (HCAECs) and induce the infarct border zone of coronary angiogenesis in a rat model of myocardial infarction [34]. Many clinical trials have shown that VEGF-based angiogenesis therapy can improve cardiac perfusion and angina class compared with placebo [35, 36]. Despite the great promise of angiogenesis gene therapy, previous studies have limited efficacy. Current randomized controlled trials have not met promising results [37]. This may be due to the low dose of agents in the local tissue of the heart and the composition of the single agent.

Actually, salidroside has been shown to promote angiogenesis significantly. It has been.reported that salidroside can increase the expression of VEGF and NO, thus promoting the angiogenesis mediated by bone marrow-derived endothelial progenitor cells [38]. Salidroside upregulates HIF-1α expression and enhances its transcriptional activity to VEGF, thereby upregulating VEGF expression at the mRNA and protein levels and finally enhancing angiogenesis and bone formation [39]. In addition, salidroside can increase the microvessels in the infarction boundary area, reduce the area of myocardial infarction and improve cardiac function in myocardial infarction mice [40]. Our molecular docking results showed that salidroside bound to several angiogenic-related factors, among which *VEGF, HIF1A* and *KDR* were correlated with coronary artery collateral circulation. In addition, cell experiments also confirmed that salidroside can promote the proliferation and migration of HUVECs. In conclusion, we believe that salidroside plays an important role in angiogenesis and collateral circulation in CAD. Due to the limited efficacy of proangiogenic therapy, salidroside as a complementary therapy may play a synergistic role in promoting angiogenesis in CAD.

Our study also has some limitations. First, we only tested the effect of salidroside on the proliferation and migration of HUVECs, even though some literatures also adopted the same method [41, 42]. However, the effect of salidroside on coronary collateral formation needs to be further verified by coronary endothelial cells and in vivo experiments. Second, even if the potential target of salidroside in promoting angiogenesis was found in this study, PCR or Western blot or surface plasmon resonance technology should be used to further verify the results in the follow-up study.

## Conclusions

Overall, in this study, the pharmacological mechanism of salidroside in CAD was investigated using network pharmacology and experimental methods. Salidroside may improves angiogenesis and collateral circulation in patients with CAD by regulating VEGF, HIF1A and KDR and other angiogenesis-related factors. However, the final mechanism needs to be further verified by subsequent experiments.

## Supplementary Information

**Additional file 1:** **Supplementary Table 1.** Targets of salidroside, CAD, and salidroside in the treatment of CAD.

**Additional file 2:** **Supplementary Table 2.** Enrichment analysis of salidroside-related targets and targets for treatment of CAD.

**Additional file 3:** **Supplementary Table 3.** Twenty-five gene sets and genes related to angiogenesis in MSigDB.

## Data Availability

The datasets generated and/or analysed during the current study are publicly available. The database links used in this article are as follows: PubChem database (https://pubchem.ncbi.nlm.nih.gov/), TCMSP database (https://tcmsp-e.com/), the Encyclopedia of Traditional Chinese Medicine (ETCM, http://www.tcmip.cn/ETCM/), similarity ensemble approach (SEA, https://sea.bkslab.org/), STITCH database (http://stitch.embl.de/), Gene Expression Omnibus database (GEO, https://www.ncbi.nlm.nih.gov/geo/), DisGeNET (https://www.disgenet.org/), DrugBank (https://www.drugbank.com/), GeneCards (https://www.genecards.org/), The Human Phenotype Ontology (HPO, https://hpo.jax.org/app/), NCBI-gene (https://www.ncbi.nlm.nih.gov/gene/), Online Mendelian Inheritance in Man (OMIM, https://omim.org/), PharmGKB (https://www.pharmgkb.org/), The CellMarker database (http://xteam.xbio.top/CellMarker/), Molecular Signatures Database (MSigDB, https://www.gsea-msigdb.org/gsea/msigdb), Protein Data Bank (http://www.rcsb.org/pdb/), and Metascape (https://metascape.org).

## Abbreviations

CAD: Coronary artery disease
GO: Gene ontology
KEGG: Kyoto encyclopedia of genes and genomes
DO: Disease ontology
CFI: Coronary flow index
HUVEC: Human umbilical vein endothelial cell
PCI: Percutaneous coronary intervention
CABG: Coronary artery bypass grafting
OB: Oral bioavailability
Caco-2: Caco-2 permeability
BBB: Blood–brain barrier
UniProt: Universal protein resource
PPI: Protein–protein interaction
MSigDB: Molecular signatures database
OD: Optical density
VSMCs: Vascular smooth muscle cells
VEGF: Vascular endothelial growth factor
